# Giant adenoid cystic carcinoma of the sinonasal cavity

**DOI:** 10.11604/pamj.2015.21.82.5833

**Published:** 2015-06-02

**Authors:** Mohamed Mliha Touati, Haddou Ammar

**Affiliations:** 1 ENT Department, Military Hospital Avicenna, Marrakech, Morocco

**Keywords:** Sinonasal cavity, adenoid cystic carcinoma, ethmoïdomaxillonasal tumor

## Image in medicine

Adenoid cystic carcinomas, formerly known cylindromas, were originally described by Foote and Frozell 1953. These are epithelial malignancies that develop at the expense of salivary glands, other rare localizations have been described in particular in the oral cavity, the sinonasal tract, lacrimal glands or nasopharynx. Clinical and radiological signs are not specific, and are common to all sinonasal tumors, including squamous cell carcinomas and adenocarcinomas. The adequate treatment is surgery, postoperative radiation therapy improves long-term prognosis. These tumors are characterized by aggressiveness and a high incidence of local recurrence and distance metastasis regardless of therapeutic modalities used. We report a case of a 70 year old patient, hospitalized for exploration of a large sinonasal tumors, CT Scann of the face and sinonasal cavities disclosed a large right ethmoïdomaxillonasal tumor, with tissue density with and contrast enhancement, lysing all the walls of the maxillary sinus, septum and the horizontal branch of the mandible. With extension to the infratemporal fossa, the orbital floor, the contralateral nasal cavity, the floor of the nasal cavity and nasopharynx. A nasal biopsy concluded to an adenoid cystic carcinoma, The staging revealed no distant metastases, the tumor was classified T4NoMo. The patient refused any surgery, he was referred for palliative radiotherapy, it was lost sight of.

**Figure 1:**
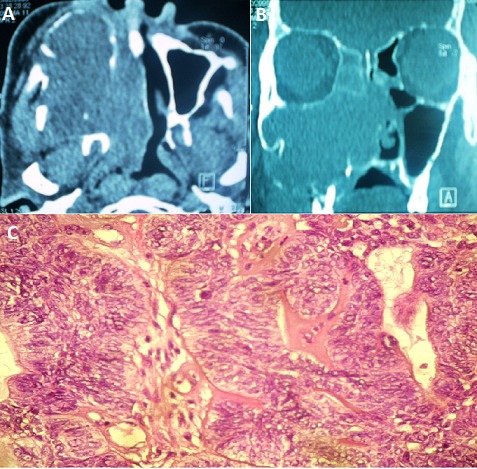
(A): computed tomography of the sinonasal area, axial section with contrast medium injection, showing a tumor process the right maxillary sinus with extension to the nasal cavity and the infatemporale fossa and bone erosion, (B): computed tomography of the sinonasal area, coronal section showing a large ethmoidomaxillar tumor process extended to the nasal cavity with lysis of intersinusonasal wall and nasal septum, (C): adenoid cystic carcinoma: cribriform lobules composed of somewhat irregular cells scattered in a fibrohyaline stroma. HE x100

